# Natural history of Krabbe disease – a nationwide study in Germany using clinical and MRI data

**DOI:** 10.1186/s13023-020-01489-3

**Published:** 2020-09-10

**Authors:** Sarah Isabel Krieg, Ingeborg Krägeloh-Mann, Samuel Groeschel, Stefanie Beck-Wödl, Ralf A. Husain, Ludger Schöls, Christiane Kehrer

**Affiliations:** 1 Department of Child Neurology, Children’s Hospital, University of Tübingen, Hoppe-Seyler-Str. 1, 72072 Tübingen, Germany; 2 Section for Experimental MR of the CNS, Department of Child Neurology and Neuroradiology, University of Tübingen, Tübingen, Germany; 3 Department of Medical Genetics, University Hospital Tübingen, Tübingen, Germany; 4 Department of Neuropediatrics, Jena University Hospital, Jena, Germany; 5 Clinical Neurogenetics Section, Department of Neurology, University of Tübingen, Tübingen, Germany

**Keywords:** Krabbe disease, Globoid cell leukodystrophy, GALC deficiency, Natural disease course, MRI pattern, Pattern recognition

## Abstract

**Background:**

Krabbe disease or globoid cell leukodystrophy is a severe neurodegenerative disorder caused by a defect in the *GALC* gene leading to a deficiency of the enzyme ß-galactocerebrosidase. The aim of this work was to describe the natural disease course covering the whole spectrum of the disease.

**Methods:**

Natural history data were collected with a standardized questionnaire, supplemented by medical record data. We defined different forms of the disease according to Abdelhalim et al. (2014). Developmental and disease trajectories were described based on the acquisition and loss of milestones as well as the time of first clearly identifiable symptoms and needs such as spasticity, seizures and tube feeding. MRI was assessed using the scoring system by Loes et al. (1999) and in addition a pattern recognition approach, based on Abdelhalim et al. (2014).

**Results:**

Thirty-eight patients were identified, from 27 of these patients 40 MRIs were available; 30 (79%) had an infantile onset, showing first symptoms in their first year of life, almost all (27 out of 30) starting in the first six months. A later onset after the first year of life was observed in 8 patients (21%, range 18 months to 60 years). Irritability, abnormalities in movement pattern as well as general developmental regression were the first symptoms in the infantile group; disease course was severe with rapid progression, e.g. loss of visual fixation, need for tube feeding and then an early death. Gait disorders were the first symptoms in all patients of the later onset groups; progression was variable. The different forms of the disease were characterized by different MRI patterns (infantile: diffuse white matter involvement and cerebellar structures specifically affected, later onset: parieto-occipital white matter and splenium affected, adult: motor tracts specifically affected).

**Conclusion:**

This is the first description of the natural history of Krabbe disease in a larger European cohort using developmental, clinical and MRI data. We would like to highlight the very different clinical and MRI characteristics of the later onset forms. These data are important for counselling affected patients and families and may serve as a basis for future treatment trials.

## Background/introduction

Krabbe disease (KD) or globoid cell leukodystrophy (GLD) is a rare autosomal recessive storage disease due to mutations in the *GALC* gene leading to a deficiency of the enzyme ß-galactocerebrosidase [21]. Krabbe disease belongs to the group of sphingolipidoses.

Most publications on the natural history of KD are on the infantile form which is also called ‘classic’ and is often subdivided into an early- and late-infantile form of Krabbe disease, with onset before or after 6 months of age [9, 27]; progression in both forms is described as rapid and rather homogenous. While older data, e.g. by Wenger et al. [28], assume that more than 90% of Krabbe patients suffer from the early-infantile form, newer data by Duffner et al. [5] indicate that 62% of the Krabbe patients show the early-infantile and 10% the late-infantile form; they describe, in addition 22% of patients with later onset and 5% with an adolescent/adult form. Slower progressive forms with a later onset are less well known and poorly systematically investigated, and may be underestimated [4, 5, 19].

In previous studies on KD, different age limits were applied in defining the different forms of disease, especially concerning patients beyond the first year of life, which makes comparability of results difficult [2, 9, 15]. Abdelhalim et al. [1] suggested a classification into five forms: early-infantile (onset 0–6 months), late-infantile (7–12 months), later onset (13 months to 10 years), adolescent (11–20 years) and adult (from 21 years on).

Until now, more than 140 different pathogenic variants of the *GALC* gene have been described [19]. The 30 kb-deletion (c.1161 + 6532_polyA+9kbdel) is with about 45 percent of the mutated alleles by far the most common mutation in the Caucasian population [6, 22]. This mutation has been reported to result in the infantile form if homozygous or compound heterozygous together with another severe mutation [22]. The mutation c.857G > A (p.Gly286Asp) in exon 8, which results in an amino acid exchange from glycine to aspartate, has been reported to lead to a disease course with a later onset if occurring homozygous or compound heterozygous together with another mutation [22]. Regional differences in the frequency of different mutations were described [18]; mutation profile, for example, is fundamentally different between European and Japanese patients [23]. In rare cases, a mutation or deletion in the PSAP gene, causing deficiency of activator protein saposin A, is reported to lead to a later onset form of KD [17, 20]. Although there is a certain relationship between genetic modifications and disease onset, genotype-phenotype-correlation is often difficult, and a reliable prediction of the disease course seems currently not possible.

Diagnosis via measurement of GALC enzyme activity presents certain challenges, even when using a radio-labelled natural enzyme substrate [13]. And pathologically reduced enzyme activity has to be distinguished from low activity found in clinically healthy people due to pseudodeficiency (usually below 15% of normal level) [29].

A hall mark of the disease is demyelination of the CNS and PNS, and magnetic resonance imaging (MRI) is an important diagnostic feature and biomarker for the description of the CNS pathology of KD [26, 32]. Distinct MRI abnormalities have been described with progressive central white matter changes and typical cerebellar pathology, involving the dentate nucleus especially in early onset forms [1]. A score has been suggested to quantify MRI-changes [14]. MRI in slower progressive forms with a later onset has been reported to show a different pattern, involving mainly supratentorial white matter [14, 22, 25]

HSCT is currently the only clinically available treatment option. Most data are on presymptomatically transplanted children, siblings of children with the infantile form of the disease [8]. In later onset forms, transplantation may be successful, when done very early during disease course [19].

Previous analyses in larger patient cohorts are based on US-American data and are focused on neurological symptoms. In Europe, to our knowledge, there is no systematic analysis of a larger number of patients with KD. The aim of this study, therefore, was the description of the natural disease course in a larger patient cohort recruited on a national basis in Germany covering the whole spectrum of the disease and focusing on developmental features, the patterns of first symptoms and MRI abnormalities at onset and during disease course.

## Methods

The German leukodystrophy network LEUKONET, funded between 2003 and 2011 was the basis for the acquisition of the first patient data, which was then continued until 2017.

The diagnosis was based on enzyme deficiency measured in leukocytes, clinical abnormalities, and leukodystrophic alterations in the MRI. In part of cases, the diagnosis was confirmed by a molecular genetic analysis of the *GALC* gene. None of the patients included in the study was diagnosed presymptomatically, e.g. based on genetic diagnostics in case of an affected sibling. Patients with all disease forms of KD were included. As this study aimed at describing the natural history of the disease, no data was collected following therapy.

Main data source was a standardized questionnaire used for leukodystrophies on birth, family history, diagnosis, motor function, language, cognition and communication as well as neurological status, clinical parameters and social-pediatric aspects [10, 12]. Medical records and parent’s documentation on the child’s disease development complemented the questionnaire data.

**Fig. 1:**
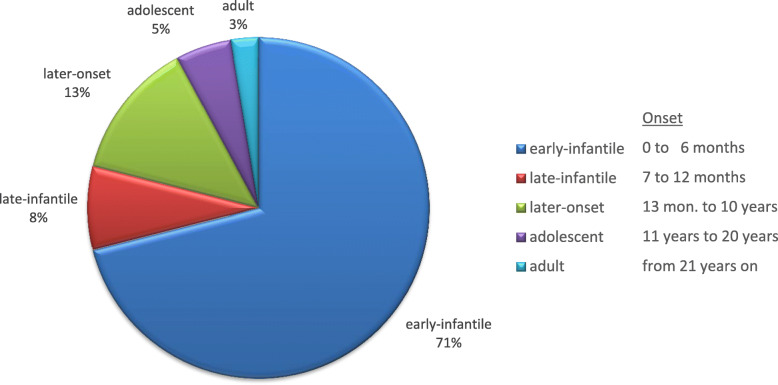
Frequency of Krabbe disease according to age at onset

We defined different forms of the disease according to Abdelhalim et al. [1] as given above (see also Fig. 1). For the standardized evaluation of gross motor function, the GMFC-MLD developed by Kehrer et al. [11] was applied.

Only MRIs done in the clinical context were used, and a standardized analysis performed. As a minimum, axial T2-weighted images were required.

Written informed consent of the patients or parents was asked for. In some rare cases, when patients and their families were known to the authors through earlier patient care, and contact could not be established anymore, available data were included without written informed consent. The study was approved by the Ethical Committee of the University of Tuebingen (no. 401/2005).

### Analysis of clinical data

A descriptive group comparison was chosen as first approach, as we expected different group sizes with less patients in the later onset groups. We considered individual disease trajectories according to the form of disease the most target-oriented method to analyze clinical and developmental features. This also takes adequate account of the missing parameters of some patients.

### Analysis of MR data

The evaluation of the MRI data was done by two child neurologists with specific experience (IKM and SG). The MRIs were first assessed according to the scoring system by Loes et al. [14] which defines ‘MR involvement as referring to any signal changes within the brain parenchyma that could be explained by GLD. Involvement can be seen as T1 hyperintensity or hypointensity, T2 hyperintensity or hypointensity, or enhancement’. Second, a pattern recognition approach was used, here changes over time were given descriptively. The latter was adopted from Abdelhalim et al. [1], who suggested four patterns (A-D), which we grouped into two categories (A1 and A2 for infantile forms, equaling his patterns A and B; and B1 and B2 for later onset forms equaling his patterns C and D). They were evaluated initially without knowledge of age at MRI and onset, the clinical parameters and the form of disease. This procedure enabled the unbiased neutral assessment of the MRIs.

## Results

From 51 patients recruited all over Germany, 38 fulfilled the inclusion criteria, from 13 patients relevant data were missing and were excluded. From 38 included patients, 17 (45%) were female. Consanguinity was present in six patients, in four, parents were first cousins. Detailed information on birth was available in 33 patients, all but one were born at term, the latter was born at 36 weeks of gestation. The developmental data in this patient were corrected for prematurity. No peri- and neonatal complications relevant for neurological outcome were reported. All clinical data referred to natural history, i.e. none of the patients had a specific treatment. Forty MRIs from 27 patients were available. However, evaluation of two MRIs was not possible because of the quality of resolution, so that 38 MRIs of 26 patients were analyzed.

Onset of the disease (Fig. 1): nearly 80% of patients (*n* = 30) had the infantile form with an onset in the first year of life, and by far the majority had an early-infantile onset (0–6 months), e.g. 27 or 71%, only 3 (7%) had a late-infantile onset (7–12 months). In around 20% of cases, the disease had an onset beyond the first year of life, in most cases (five out of 8 patients) the disease started in the first decade.

First symptoms: As shown in Table 1, first symptoms were different when onset was in the first year of life than when the disease started later. In most of the infantile cases (80%), agitation and irritability were mentioned as first symptoms; abnormalities in movement pattern and general development regression were also frequent (63%). Feeding problems in 9 (24%), weakness in 4 (11%) and pain in 4 (11%) patients were also mentioned as additional first symptoms. In all patients with onset beyond the first year of life, first signs concerned abnormalities of movement patterns which manifested as a gait disorder. Abnormalities in fine motor skills and a general development regression were also often mentioned as first symptoms (63 and 50%). In addition, behavioral disorders were mentioned in 10 (26%), restlessness in 27 (70%), and pain in one patient. In our two adolescent patients, problems of concentration were also mentioned.

**Table 1:** Absolute and relative frequencies of first symptoms which led to diagnostic identification

**Infantile onset (0 to 12 months)**		
1. Agitation/Irritability	24/30	80%
2. Abnormalities in movement pattern and general development regression	19/30	63%
**Onset beyond the first year of life**		
1. Gait disorder/Abnormalities in movement pattern	8/8	100%
2. Abnormalities in fine motor skills	5/8	63%
3. General development regression	4/8	50%

### Description of the natural history based on the acquisition and loss of milestones

#### Early-infantile patients

**Fig. 2:**
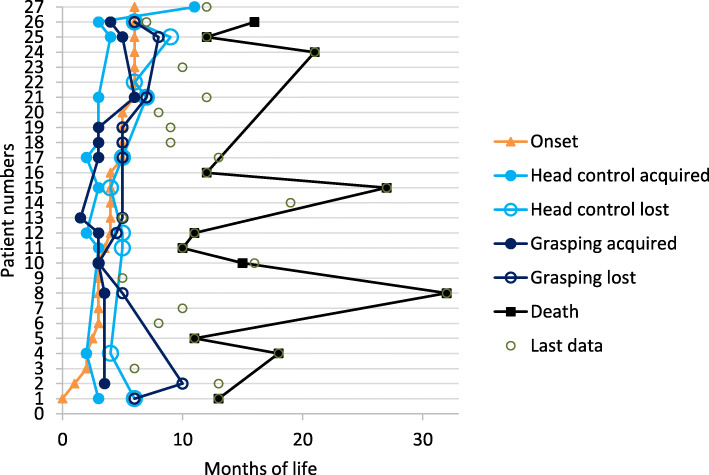
Gross and fine motor development and disease course of the early-infantile group. Categories are represented by symbols. The symbols for each category are linked together for better identification.

Figure 2 shows the acquisition and loss of motor milestones. Age at onset ranged between 0 and 6 months (mean 4 months): Around half of the patients acquired head control (13 out of 27, in 5 patients it was unknown whether acquired), which was lost again within a short time (on average, 1.5 months after onset head control was lost again). Data on grasping were available in 21 out of 27 patients: 13 (48%) had learnt to grasp, and lost it again soon thereafter, on average about 2 months after onset. In 12 patients we know the time of death, which showed a certain variability (between 10 and 32 months, mean 13 months). Earlier onset was not consistently related to earlier age at death.

**Fig. 3:**
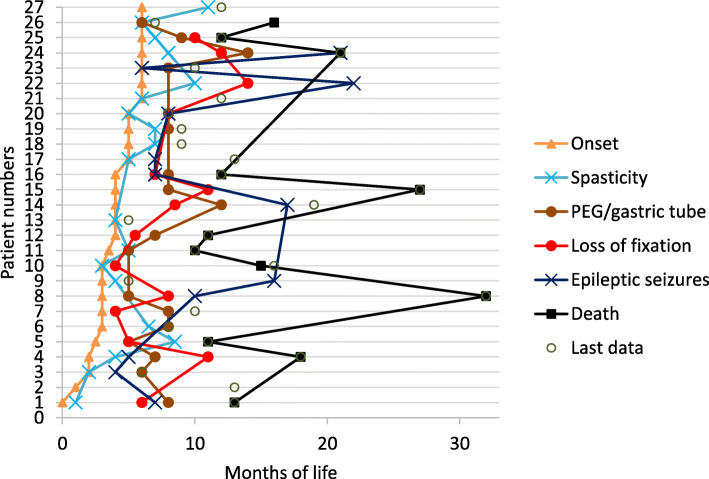
Onset of neurological symptoms in the early-infantile group

The age at onset of neurological symptoms such as spasticity, epileptic seizures and loss of fixation, as well as the time of insertion of a gastric tube is shown in the early-infantile patients with respect to disease onset in Fig. 3. Spasticity is a very early feature, appearing at or briefly after onset. Loss of fixation occurs rather early, on average about 4.5 months after onset. Severe swallowing problems necessitating tube feeding or PEG occurred on average 4 months after onset. Occurrence of seizures was more variable. In all but one patient (in whom parents had observed single words) no language production was reported.

### Late-infantile patients

The late-infantile group comprised only 3 patients. First symptoms appeared at 9, 9 and 10.5 months. Initial symptoms were abnormalities in movement pattern (3), agitation/irritability (2), abnormalities in fine motor skills (2), general development regression (2) and feeding problems (1). The disease progression was rapid, loss of head control occurred at a mean after 3.5 months, loss of fixation after 7 months, spasticity was first observed 3 months, epileptic seizures 3 months, hearing impairment 11 months, swallowing problems 3 months and mucous obstruction 7 months after onset. Thus, similar as in the early-infantile group, the late-infantile patients showed a rapid progression of disease with a loss of milestones as well as the occurrence of neurological symptoms within the first year after onset.

### ‘Later onset’ group

**Fig. 4:**
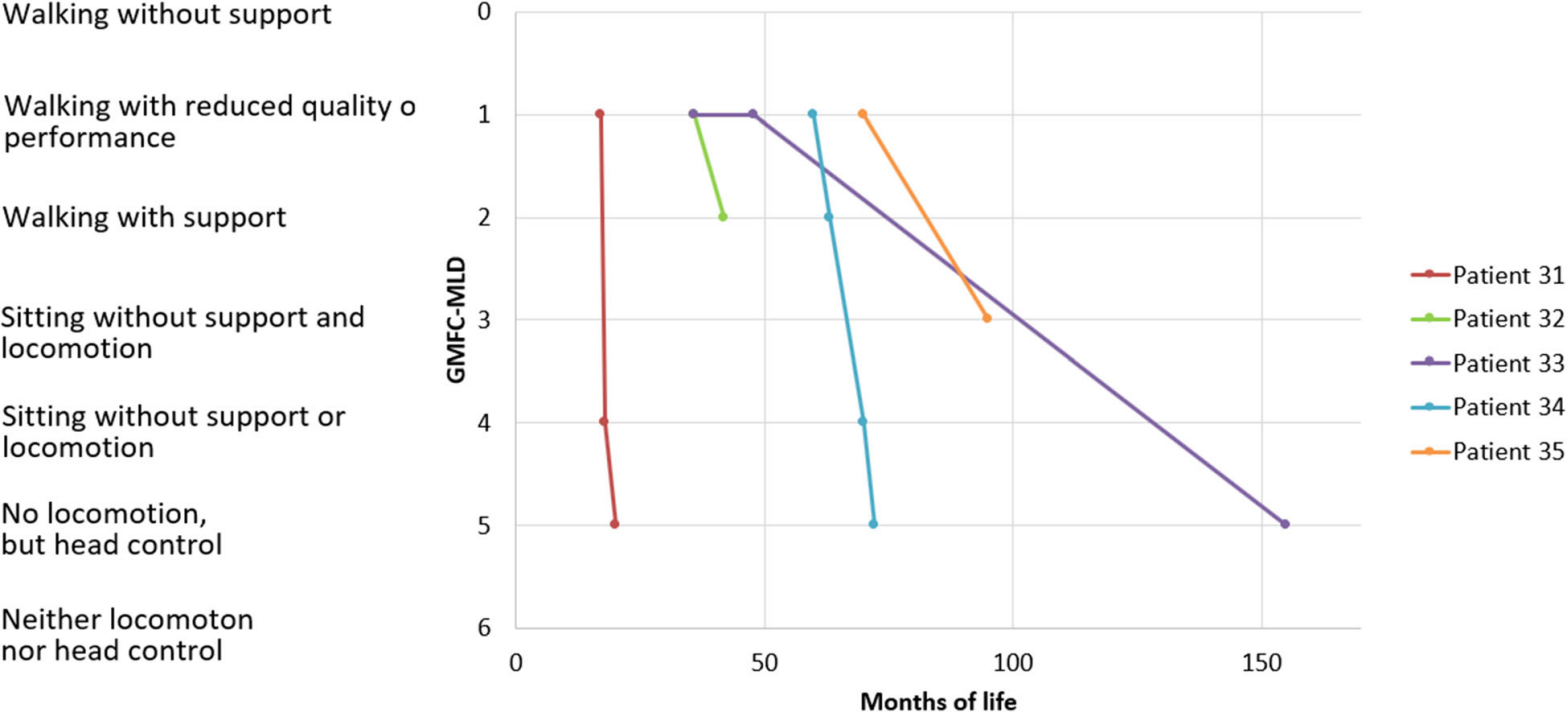
Individual courses of gross motor function according to the GMFC-MLD

Figure 4 displays the gross motor function of patients in this group as assessed with the GMFC-MLD-score. All ‘later onset’ patients had a normal development with regular acquisition of milestones until the onset of symptoms which were gross motor abnormalities in all cases. Two patients (pat. 31 and 34) with an onset more than 3 years apart showed a rapidly progressive deterioration with a loss of all gross motor function except head control within two and twelve months respectively, while a patient with an onset between the two (pat. 33) had a clearly slower course.

### Adolescent/adult group

Our cohort included two patients with an adolescent (12 and 19 years) and one with an adult (60 years) onset. In our adolescent patients, concentration problems had been reported prior to gross motor symptoms for many years; they were, however, ill defined and it seemed unlikely that they were really due to Krabbe disease. In all three patients gross motor abnormalities presenting as gait disorders were the clear initial symptoms. Clinical courses are known until 4, 10 and 11 years after onset, when the adolescent patients were still able to walk without support, whereas the adult patient lost this ability 8.5 years after onset.

### Evaluation of MRI

From 38 evaluated MRIs, 25 were from early-infantile, 5 from late-infantile, 6 from later onset, and 2 from adolescent/adult patients. In 9 patients we could evaluate MRI alterations in the disease course as we had more than one MRI from these patients. Data of the Loes score are detailed in.

**Fig. 5:**
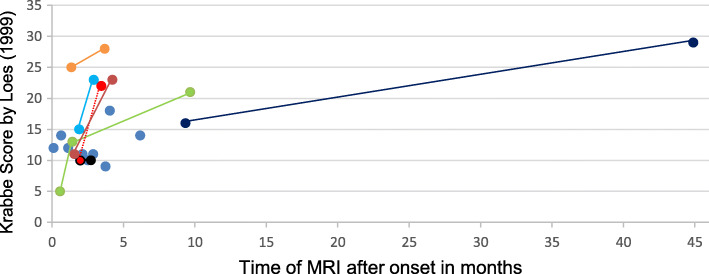
Development of MRI abnormalities (Loes score) during disease course of the early-infantile form

Table 2 and give specific information on structures affected. MRIs of the early-infantile patients assessed with the Loes Score: The score increased rapidly during disease course (Fig. 5). The example of a case highlighted in dashed line showed an increase from 10 to 22 points within only six weeks.

**Table 2:** First MRIs compared to follow-up MRIs in the disease course of all groups

Patient number	Age at onset (m)	Time MR after onset (months).	Parieto occipital wm				Anterior temporal wm				Frontal wm				Corpus callosum					Visual pathway				Pyram syst			Cerebellum			Basal ganglia	Ant. thalamus	Atrophy			Loes Score
			Periventr.	Central	Subcortic.	Atrophy	Periventr.	Central	Subcortic.	Atrophy	Periventr.	Central	Subcortic.	Atrophy	Splenium	Body	Genu	Atr. Sple.	Atr. Genu	Optic rad.	Mey. loop	Lat gen b.	Optic tract	Cor. rad.	Int caps.	Brain stem	WM	Den. nuc.	Atrophy			Mild	Moderate^1^	Severe^1^	
**First MRI early-infantile**																																			
1	0	6	●	●							●	●			●	●	●							●	●	●	●	●			●	●			14
4	2	2	●	●							●	●				●								●	●		●	●			●				10
5	2,5	2	●	●							●	●			●	●								●			●	●			●				10
6	3	3	●	●							●	●				●								●	●		●	●			●	●			11
8	3	1,5	●	●							●	●			●	●								●	●		●	●			●				11
12	4	1,5	●	●	●		●	●	●		●	●	●		●	●	●	●	●	●	●			●	●	●	●	●			●	●	●	●	25
13	4	0,5									●	●												●				●			●				5
14	4	4	●	●			●	●				●			●	●	●			●	●			●	●	●	●	●	●		●	●			18
16	4	1	●	●							●	●				●								●	●	●	●	●			●	●			12
17	5	3,5	●	●							●					●								●		●	●	●			●				9
18	5	2	●	●							●	●			●	●	●							●			●	●			●				11
19	5	2	●	●							●	●			●	●	●							●	●	●	●	●			●	●			15
22	6	9	●	●							●	●			●	●	●			●	●			●	●	●	●	●				●			16
23	6	0,5	●	●							●	●			●	●	●							●	●	●	●	●			●	●			14
25	6	2,5	●	●							●	●			●	●	●							●	●			●							10
26	6	0	●	●							●	●			●	●								●	●	●	●	●			●				12
27	6	6,5	●	●		●	●				●	●			●	●	●	●		●	●			●	●	●	●	●			●	●	●		20
Follow-up MRIs early-infantile																																			
4	2	3,5	●	●	●		●	●			●	●	●		●	●	●	●	●	●	●			●	●	●	●	●			●	●			22
5	2,5	2,5	●	●							●	●			●	●								●			●	●			●				10
8	3	4	●	●	●		●	●	●		●	●	●		●	●	●	●	●	●	●	●		●	●	●	●	●			●	ne	ne	ne	23
12	4	3,5	●	●	●	●	●	●	●	●	●	●	●	●	●	●	●	●	●	●	●			●	●	●	●	●			●	●	●	●	28
13	4	1,5	●	●							●	●			●	●	●							●	●	●		●			●	●			13
13	4	9,5	●	●	●	●				●	●	●	●	●	●	●	●	●	●	●				●	●	●	●				●	●			21
19	5	3	●	●	●		●	●			●	●		●	●	●	●			●	●			●	●	●	●	●		●	●	●	●	●	23
22	6	45	●	●	●	●	●	●	●	●	●	●	●	●	●	●	●	●	●	●	●	●		●	●	●	●	●	●			●	●	●	29
**First MRI late-infantile**																																			
28	9	2,5	●	●							●	●				●								●	●	●									8
30	10,5	1	●	●							●	●			●	●								●	●	●					●	●			11
Follow-up MRI late-infantile																																			
28	9	3,5	●	●	●						●	●				●								●	●	●									9
28	9	4	●	●	●						●	●			●	●								●	●	●									10
28	9	10	●	●	●		●	●	●		●	●	●		●	●	●			●	●	●		●	●	●	●		●			●			21
**First MRI later onset**																																			
31	17	1	●	●			●	●			●	●			●	●				●	●			●											11
32	36	4	●	●			●	●				●						●		●	●			●	●										10
33	36	118	●	●	●	●					●	●	●		●	●		●						●	●	●									13
34	60	2	●	●	●	●					●	●			●	●		●		●				●	●	●									13
35	70	5	●	●	●	●	●	●			●	●	●		●	●		●		●				●	●							●			16
Follow-up MRI later onset																																			
34	60	3	●	●	●	●					●	●			●	●		●		●				●	●	●									13
**First MRI adolescent/adult**																																			
36	144	16	●	●			●								●					●	●				●	●									8
38	720	115		●								●	●											●	●				●			●			7

● – Structure affected, empty cell – Structure not affected, ^1^ Atrophy: Cumulation of points, ne – Structure could not be evaluated

*WM* White matter

This score is very valuable to show the dynamic of MR changes. However, it does not give the different MR pattern of the onset form, which is essential for diagnosis. Therefore, a pattern recognition analysis was done in addition.

**Fig. 6:**
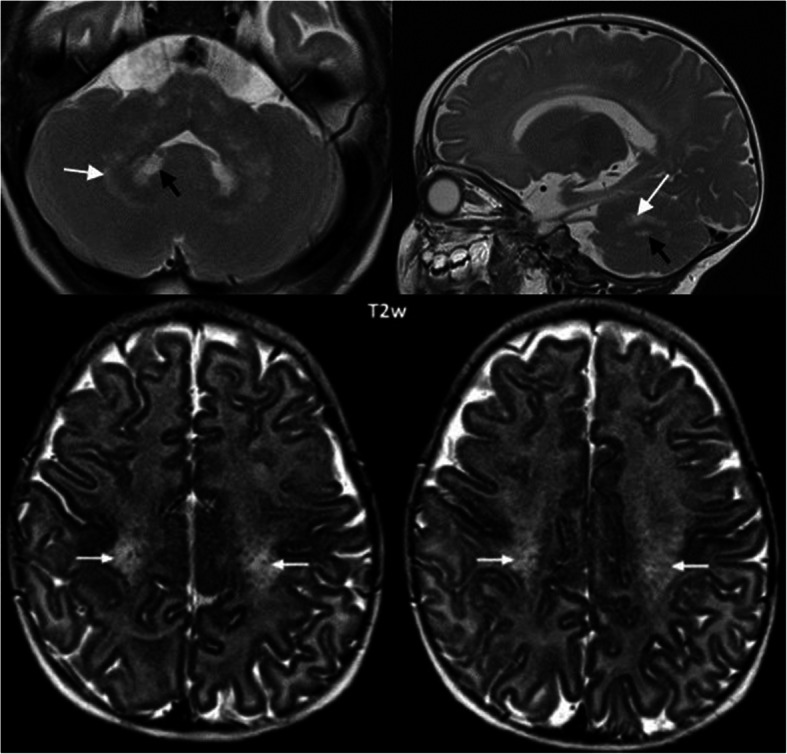
Pattern A1 (early-infantile form). White matter changes prominent in the central region (small white arrows in lower images), cerebellar white matter (white arrows in upper images) and signal changes of the dentate nucleus (black arrows in upper images). T2w images axial lower part, coronal upper left and sagittal upper right. Patient 4 (onset 2 months, age at MRI 5 months).

The pattern recognition analysis was used to describe the entire group:

Pattern A1 shows a combination of cerebral white matter changes, beginning in the central region, cerebellar white matter changes and signal changes of the dentate nucleus, as well as an involvement of the Corpus callosum and the pyramidal tract (Fig. 6). All MRIs of the early-infantile patients displayed this pattern.

**Fig. 7:**
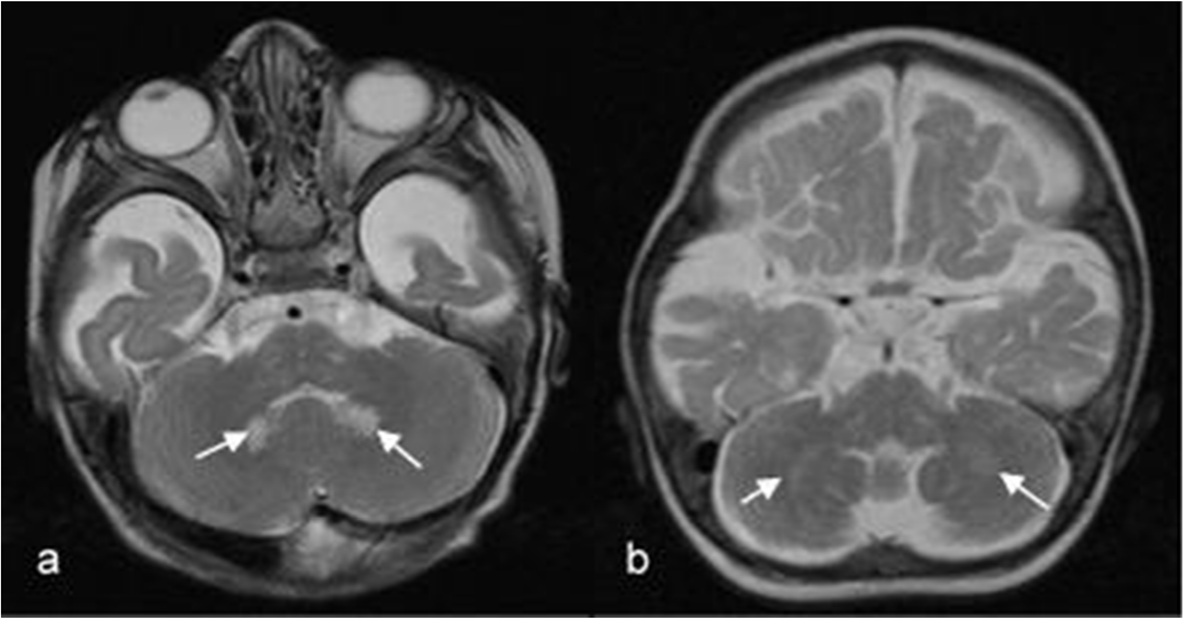
Pattern A1: cerebellar changes in an early-infantile patient during the disease course (onset 4 months). The T2-weighted axial MRI at age 5.5 months shows hyperintensity in the dentate nucleus (**a**) which has disappeared eight months later (**b**).

MRI changes during disease course (pattern A1):

In the early-infantile form, signal change in the dentate nucleus (hyperintense on T2w) was an early sign, which was no longer visible some months later (see Fig. 7).

**Fig. 8:**
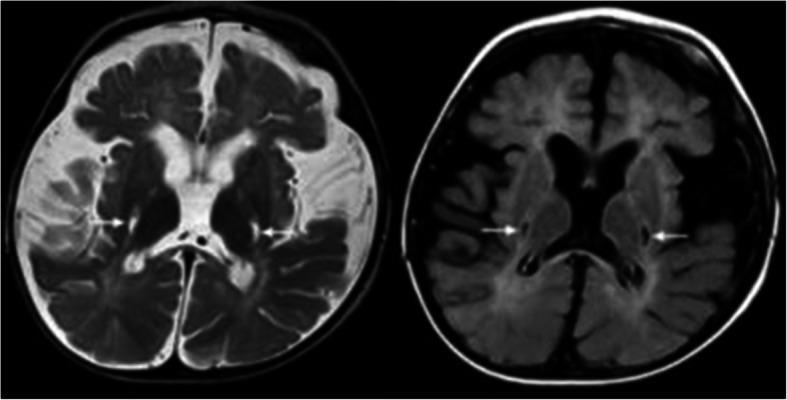
Pattern A1 (early-infantile form) at an advanced stage. Axial images (T2w left, Flair right) show cystic degeneration of the pyramidal tract (white arrows), severe atrophy with enlarged insulae and diffusely affected white matter. Patient 12 (onset 4 months, age at MRI 8 months).

In the advanced stage of the disease, cystic degeneration of the pyramidal tract, global atrophy and a diffusely affected white matter were seen (Fig. 8).

**Fig. 9:**
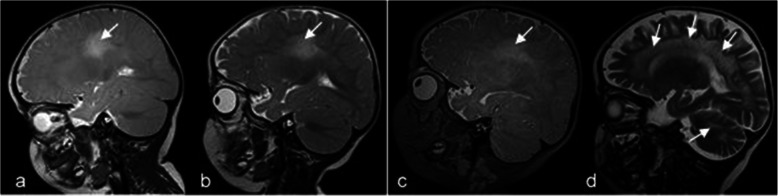
Pattern A2: MR images of a late-infantile patient in the disease course. Patient 28 (onset 9 months). At 11.5 months, the central white matter does, while cerebellar white matter does not show abnormalities (**a)**. One and 1 ½ months later, parieto-occipital white matter is increasingly involved, the cerebellar white matter still not affected (**b**,**c**). Six months later, frontal, central and parieto-occipital white matter as well as cerebellum are affected (**d**).

Pattern A2 is very similar to pattern A1, only the dentate nucleus is not affected (Fig. 9). This pattern was seen in two children with a late-infantile onset.

MRI changes during disease course (pattern A2):

Cerebellar white matter seems to be affected relatively late during disease course and demyelination starts in the central region and spreads forwards and backwards as illustrated in Fig. 9: the four T2-weighted sagittal MRIs show the disease course over 8 months, illustrating a severe progression.

**Fig. 10:**
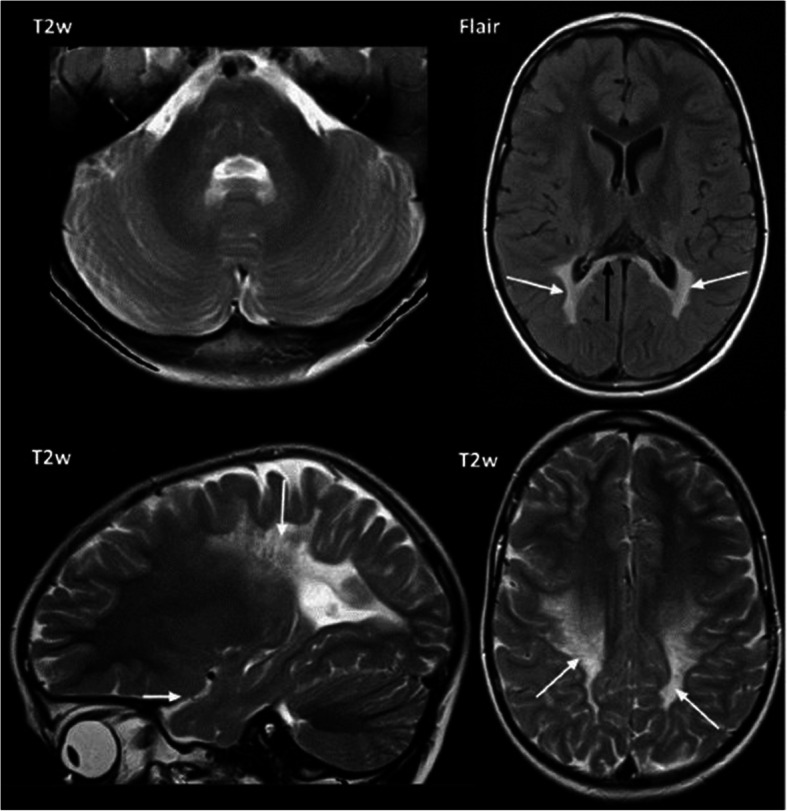
Pattern B1: MR images of a ‚later onset‘patient. Patient 34 (onset 5 years, age at MRI 5 years 1 month). White matter is primarily affected in the periventricular parieto-occipital regions (arrows in the upper right Flair image, and lower left sagittal and lower right T2w images). The Flair image shows the involvement of the splenium of the corpus callosum (black arrow). Cerebellar white matter is not affected (axial T2w image upper left).

In pattern B1, the white matter shows signal changes primarily in the periventricular parieto-occipital region. Corpus callosum is affected in body and splenium. In addition, we could see abnormalities in the pyramidal tract as in pattern A1 and A2. The cerebellum is not affected (Fig. 10).

This pattern B1 was seen in all later onset patients, i.e. all patients with first symptoms after the first year of life and up to the age of ten years (pat. 31–35). One patient with late-infantile onset (pat. 30, first symptoms at 10.5 months) and one patient with onset beyond 10 years (pat. 36, first symptoms at 12 years) showed this pattern. It seems of interest, that MRI of patient 33 with a relatively slow course was done rather late (118 months after onset) and had a Loes score of 13, which was similar to the score in the other four patients, where MRI was done much earlier (Table 2).

**Fig. 11:**
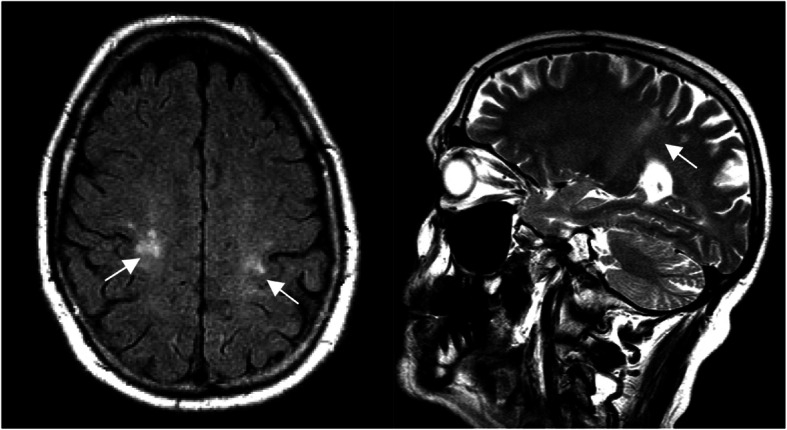
Pattern B2: MR image of an adult patient. Relatively isolated signal changes of the pyramidal tract (arrows, T2w sagittal left, axial Flair right). The cerebellum shows no signal changes. Patient 38 (onset: 60 years, age at MRI: 69 years).

In pattern B2 abnormalities are almost only visible in the pyramidal tract. Some focal abnormalities are also seen in the white matter (Fig. 11). We saw this pattern in an adult patient with a late onset, who was clinically mildly affected with a gait disorder at the time of this MRI (pat. 38, first symptoms at 60 years).

Genetic results were available in 13 out of 38 patients (7 early-infantile, 1 late-infantile, 3 later onset, 1 adolescent, 1 adult) (see Table 3). The pathogenic variant (c.1161 + 6532_polyA+9kbdel) associated in the literature to infantile onset [16] was present in four patients with an infantile onset (homozygous in one and compound heterozygous in three, two showing the same combination with the pathogenic variant c.1586C > T [31]). It also occurred in patients with an onset beyond one year: one had an adolescent onset and showed compound heterozygosity with a pathogenic variant (c.1829A > C), which has been described in the literature in two patients with a later onset [3]; in one patient with adult onset the second pathogenic variant could not be found.

**Table 3:** Onset, genotype and MRI pattern of all Krabbe disease forms

Pat. no.	Onset in months	Genotype						Age at first MRI in months	MRI Pattern
		Allele 1c	Allele 1p	Allele 2c	Allele 2p	Heterozygous father	Heterozygous mother		
**Early-infantile**									
1	0							6	A1
2	1								
3	2								
4	2	**c.1161 + 6532_polyA + 9kbdel**		c.1586C > T	p.Thr529Met			4	A1
5	2,5							4,5	A1
6	3							6	A1
7	3	c.908C > T	p.Ser303Phe	c.908C > T	p.Ser303Phe	Consanguine parents		4,5	1
8	3							4,5	A1
9	3								
10	3								
11	3,5								
12	4	c.966delA	p.Cys322Trpfs*	c.1901 T > C	p.Leu634Ser			5,5	A1
13	4							4,5	A1
14	4							8	A1
15	4								
16	4	**c.1161 + 6532_polyA + 9kbdel**	p.Arg168Cys	c.905C > G	p.Thr302Ser	yes (point mutation in exon 9)	yes (30 kb deletion)	5	A1
17	5							8,5	A1
18	5							7	A1
19	5	c.453G > A	p.Trp151*	c.1586C > T	p.Thr529Met	yes (c.1586C > T)	yes (c.453G > A)	7	A1
20	5								
21	6								
22	6							10	A1
23	6							6,5	A1
24	6	*c.430delA*	p.lle144Leufs*27	c.1186C > T	p.Arg396Trp; Exon 11	yes for mutation (c.1186C > T)	yes for deletion (c.430delA)		
25	6	**c.1161 + 6532_polyA + 9kbdel**		**c.1161 + 6532_polyA + 9kbdel**		yes (30 kb deletion)	yes (30 kb deletion)	8,5	A1
26	6							6	A1
27	6							12,5	A1
**Late-infantile**									
28	9							11,5	A2
29	9								
30	10,5	**c.1161 + 6532_polyA + 9kbdel**		c.1586C > T	p.Thr529Met	yes (30 kb deletion)	yes for mutation (c.1586C > T)	11,5	B1
**Later onset**									
31	36	c.205C > T	p.Arg69*	c.1700A > C	p.Try567Ser	results not available	results not available	18	B1
32	17	c.1627G > C	p.Ala543Pro	c.1627G > C	p.Ala543Pro	yes	yes	40	B1
33	36							154	B1
34	60							62	B1
35	70	*c.430delA*	p.Ile144Leufs*27	c.857G > A	p.Gly286Asp	yes for mutation (c.857G > A)	yes for deletion (c.430delA)	75	B1
**Adolescent and adult onset**									
36	144	**c.1161 + 6532_polyA + 9kbdel**		c.1829A > C	p.Asp610AIa	yes (30 kb deletion)	yes for mutation (c.1829A > C)	160	B1/B2
37	228								
38	720	**c.1161 + 6532_polyA + 9kbdel**		Second pathogenic variant could not be found				835	B2

^1^ MRI not assessable

## Discussion

Most publications on the natural history of KD are on the infantile form. Slower progressive forms with a later onset are less well known and only poorly systematically investigated, and are probably much rarer. Knowledge on the distinct forms is important not only for early diagnosis, patient and family counselling but also with respect to potential available therapies.

Most systematic studies on KD concerning natural history and MRI were done in non-European populations. We wanted to present such data in a larger European cohort, collected nationwide in Germany and including all age ranges.

We used the classification into five groups according to onset suggested by Abdelhalim et al. [1]. Early-infantile Krabbe was by far the most frequent form found in more than two third of patients. Together with 8% of late-infantile patients, infantile onset represents nearly 80% in our cohort, which is higher than described in the American population [5]; onset beyond the first year of life was found in around 20% with later onset representing around two-third of this group. Very few patients had an adolescent or adult onset.

As has been described before, first symptoms in infantile Krabbe were irritability and developmental regression, while all patients with an onset beyond the first year of life showed gross motor symptoms as first signs. The latter finding seems worthwhile underlining as this is different from MLD, the other classical leukodystrophy within the group of sphingolipidoses, where gross motor symptoms characterize late-infantile and early-juvenile forms, whereas in later onset forms pure cognitive and behavioral symptoms may precede gross motor symptoms for years [12].

With respect to disease course, the early-infantile form was very rapidly progressing and homogeneous. Around half of the patients had acquired head control and grasping, but lost it shortly after onset. Spasticity was seen either already at onset or shortly thereafter and loss of fixation and vision followed within around 4,5 months. Need for tube feeding was in close proximity to loss of fixation, whereas occurrence of seizures was more variable, usually later. In the children with known age at death, this occurred around 13 months after first symptoms with a certain variability, e.g. early onset was not consistently related to an earlier age at death. The disease process starts so early, that initially acquired motor function is quickly regressive and language development in terms of words is usually not possible.

Patients with a late-infantile onset showed also a rapid gross motor regression with a loss of milestones after a normal development. The later onset, adolescent and adult forms, however, appear more variable concerning progression of the disease, the latter seems correlated to age at onset only to a minor degree. But it was evident that in the adolescent and adult form, loss of milestones (loss of independent walking) can occur even several years after first gross motor symptoms (gait abnormalities).

The description of the clinical course of a rare disease is challenging. Diagnosis may be delayed so that first symptoms and first examinations are not recorded or done in expert centers. Also on follow-up, patients are seen in different centers. Thus, a standardized recording may be difficult. Reports on larger groups have centered on age at onset, first symptoms and survival [6] or have suggested a staging system based on gross signs of neurologic disease progression [7]. We chose to describe the natural history in developmental and disease related trajectories based on the acquisition and loss of milestones as well as the time of first clearly identifiable symptoms and needs such as spasticity, seizures and tube feeding. We think, these are relatively easy to assess also retrospectively, and can also be given by non-medical observers. The developmental description has proven robust and reliable also in other diseases [10, 12].

This proved to be useful especially in the infantile forms, where functional scores to describe gross motor progression of the disease, such as the GMFC-MLD [11], are not applicable due to the early onset at a young age and little gross-motor development of the patients (the GMFC-MLD is applicable beyond the 90th percentile of expected ability to walk freely from 18 months onwards). The GMFC-MLD was useful, however, to describe disease dynamics in the later onset forms.

Ideally, a generally accepted score should be available, to describe any progressive disease in comparison to healthy people [24]. Looking at Krabbe disease, however, already this single, monogenetic disease shows quite heterogeneous clinical courses representing a challenge for the common description of the whole entity. A multilayer and multidisciplinary coverage of abilities could give a comprehensive view, but would be too complex for a quick overview. Thus, describing the disease with different tools in the different forms, but with the common aim of capturing disease trajectories, seems a compromise which could be valid also for other diseases characterized by very different onset forms [2].

For the description of MRI also, combining two systems seemed useful to us. A pattern recognition approach to identify the different forms seems especially important for early diagnosis. All patients with the early-infantile form showed the typical pattern described by Abdelhalim et al. [1] and van der Knaap and Valk [25], e.g. demyelination in the central and periventricular white matter, the cerebellar white matter and the nucleus dentatus. MRI in the late-infantile form showed periventricular and primarily centrally accented demyelination similar to the early-infantile form, but with cerebellar white matter abnormalities occurring late. And compared to the early-infantile form, the late-infantile form never showed abnormalities in the dentate nucleus. Concerning onset forms beyond the first year of life, the MRI hallmark is involvement of the pyramidal tract, which can occur in isolation in the adult form or associated to demyelination in the parieto-occipital region and splenium of the corpus callosum in the later onset and adolescent forms.

We, therefore, chose to classify the patterns according to onset described by Abdelhalim et al. [1] into pattern A for the infantile groups (A1 supratentorial white matter involvement with involvement of dentate nucleus and A2 without) and B for the onset forms beyond the first year of life (B1 with more extensive involvement of posterior white matter and splenium, B2 with mainly pyramidal tract involvement). We also wanted to draw attention to pattern B as indicative of KD in its later onset, as according to our clinical experience, it is not as well recognized by the clinician in comparison to pattern A. Especially with respect to therapeutic options, this seems of importance, as efficacy of HSCT is essentially related to early timing of this therapeutic intervention [19].

A system to quantify MRI changes, as does the Loes score [14], seems very useful to illustrate the dynamic of the disease over time and is, thus, an essential tool concerning the evaluation of effects of therapy. Using it here, we could illustrate the homogeneous and extreme rapidity of the disease in the early-infantile form.

Taken together, the early-infantile form was by far the most common and also the most homogeneous in its clinical and MRI presentation and rapid progression. The late-infantile form could be seen as an intermediate form between the early-infantile form and the later onset form with respect to disease dynamics and MRI characteristics. And for the later onset form, it has to be underlined that age at onset does not seem to predict the dynamic of disease progression of the disease which seems important concerning questions of therapeutical intervention.

Looking at the different genetic mutations with respect to age at onset and the disease form respectively, suggests that the combination of the mutations on both alleles is critical. This can explain that a big deletion if homozygous leads to the severe and rapidly progressive form of an early-infantile patient, but may cause an adolescent or adult onset with a slowly progressive course if combined with another pathogenic mutation.

Furthermore, gene regulation, epigenetic factors and polymorphisms may influence the impact of mutations and make genotype-phenotype-correlation difficult. Further analysis of these factors may allow to explain why the same combination of heterozygous mutations in our cohort lead to slightly different courses, e.g. early- and late-infantile. It has been suggested that the presence of a common and per se benign variant (“polymorphism”) may influence another per se benign variant to develop into a disease causing pathogenic variant [30]. This could lead to disease manifestation if combined with a pathogenic variant on the other allele.

Considering that our knowledge of genotype up to now does not clearly allow to predict disease course, it seems all the more important to systematically study natural history with respect to standardized description of clinical and MRI features.

## Conclusion

The aim of this work was the description of the natural history of KD in a nationwide patient cohort using developmental, clinical and MRI data. To our knowledge, this is the first systematic analysis in a comparably large patient cohort of this rare disease in Europe. For the neuroimaging data, the combination of a pattern recognition and a quantification approach seems useful for the identification of different forms on the one hand and follow-up on the other.

These data are important for counselling affected patients and families and may serve as a basis for future treatment trials.

## Abbreviations

HSCT: Haematopoetic Stem Cell Transplantation
MRI: Magnetic Resonance Imaging
